# Human Epithelial Cells Discriminate between Commensal and Pathogenic Interactions with *Candida albicans*

**DOI:** 10.1371/journal.pone.0153165

**Published:** 2016-04-18

**Authors:** Timothy J. Rast, Amy L. Kullas, Peter J. Southern, Dana A. Davis

**Affiliations:** Department of Microbiology, University of Minnesota, Minneapolis, MN, United States of America; David Geffen School of Medicine at University of California Los Angeles, UNITED STATES

## Abstract

The commensal fungus, *Candida albicans*, can cause life-threatening infections in at risk individuals. *C*. *albicans* colonizes mucosal surfaces of most people, adhering to and interacting with epithelial cells. At low concentrations, *C*. *albicans* is not pathogenic nor does it cause epithelial cell damage *in vitro*; at high concentrations, *C*. *albicans* causes mucosal infections and kills epithelial cells *in vitro*. Here we show that while there are quantitative dose-dependent differences in exposed epithelial cell populations, these reflect a fundamental qualitative difference in host cell response to *C*. *albicans*. Using transcriptional profiling experiments and real time PCR, we found that wild-type *C*. *albicans* induce dose-dependent responses from a FaDu epithelial cell line. However, real time PCR and Western blot analysis using a high dose of various *C*. *albicans* strains demonstrated that these dose-dependent responses are associated with ability to promote host cell damage. Our studies support the idea that epithelial cells play a key role in the immune system by monitoring the microbial community at mucosal surfaces and initiating defensive responses when this community is dysfunctional. This places epithelial cells at a pivotal position in the interaction with *C*. *albicans* as epithelial cells themselves promote *C*. *albicans* stimulated damage.

## Introduction

*Candida albicans* is a natural isolate of the human oral-pharyngeal cavity and the gastro-intestinal and uro-genital tracks [1]. While *C*. *albicans* primarily colonizes these mucosal surfaces at low levels as a commensal, it can overgrow and cause superficial infections. For example, *C*. *albicans* is commonly present within the vaginal tract, yet most women suffer at least one episode of vulvovaginal candidiasis (VVC) and 5–10% of women suffer from recurrent VVC [2]. *C*. *albicans* can escape from mucosal sites in susceptible hosts and enter the bloodstream to cause life-threatening systemic candidiasis, which has an attributable mortality of 30–50% even with antifungal therapy [3]. Thus, the mucosal surface represents both the primary site of *C*. *albicans*-host interaction and the origin of serious clinical infections.

Mucosal surfaces are composed of one or more layers of epithelial cells and define the major interface between the environment and the host. For example, the intestinal epithelia of the ileum, jejunum, and colon have a single layer of columnar epithelial cells, whereas other mucosal tissues have layers of stratified epithelial cells. Mucosal tissues also contain resident immune cells, including dendritic cells, macrophage, and T-cells. These cells are often found in immunologic foci such as Peyer’s patches, nasopharynx associated lymphoid tissue, or oral lymphoid foci and/or are distributed throughout the sub-mucosa adjacent to the epithelial surface. Epithelial cells play a key role in signaling immune cells and the epithelial surface is an active and essential component of innate immunity. Thus, epithelial cells represent the first line of defense against *C*. *albicans* infections.

To gain a clear understanding of *C*. *albicans* pathogenesis, it is essential to understand the initial interactions between *C*. *albicans* and mucosal epithelial cells. Most studies addressing *C*. *albicans* host-pathogen interactions have focused on *C*. *albicans* in systemic infection models and have identified a plethora of *C*. *albicans* genetic requirements for pathogenesis. These studies focus on events occurring after *C*. *albicans* has escaped from the mucosal surface frequently overlook the role of the host in *C*. *albicans* host-pathogen interactions.

Several models have been developed to study the interaction between *C*. *albicans* and epithelial cells, which have led to a number of important insights. Filler and colleagues established an *in vitro* model of *C*. *albicans* mediated epithelial cell damage [4]. In this model, *C*. *albicans* yeast cells germinate to form hyphae and adhere to the epithelial cell monolayer. Epithelial cells then phagocytose portions of the hyphae, leading to *C*. *albicans*-mediated epithelial cell lysis. This model has revealed molecular details likely involved in dissemination and is key for continued understanding of *C*. *albicans* pathogenesis. For example, the *C*. *albicans* adhesin Als3 binds to epithelial cell E-cadherin and this interaction is important for epithelial cell damage [5]. Using a reconstituted epithelial cell model, Villar and colleagues found that the *C*. *albicans* secreted aspartyl protease (Sap) Sap5 degrades epithelial cell E-cadherin promoting tissue invasion [6]. *ALS3* and *SAP5* are induced by the pH responsive transcription factor Rim101 [6, 7], suggesting that Rim101 governs two interactions with the mucosal surface. In addition to providing key insights into *C*. *albicans* pathogenesis, these studies have also demonstrated that host responses are critical components of fungal-induced tissue damage. For example, inhibition of host cell phagocytosis of *C*. *albicans* hyphae completely blocks host cell damage [8].

While recognition and understanding the host side of the *C*. *albicans*-epithelial cell interaction is still in its infancy, studies with other pathogenic microbes have demonstrated the key roles epithelial cells play in innate immunity. Epithelial cells sense environmental microbes using a set of pattern recognition receptors (PRRs), such as the Toll-like receptors (TLRs), which recognize common microbial motifs. TLR2 and TLR4 have been implicated in sensing *C*. *albicans* [9, 10]. Activated PRRs stimulate downstream MAP kinases, such as ERK1/22, JNK1/2, and p38, which phosphorylate and activate transcription factors, including AP-1 or CREB, inducing immune and inflammatory responses, like expression of IL-8. These inflammatory responses activate resident immune effector cells, such as macrophages, and recruit additional effector cells, including neutrophils, to sites of infection. Thus, epithelial cells are poised at the front line to sense and respond to *C*. *albicans* cells at the mucosal surface.

Many of the previous studies of *C*. *albicans*-epithelial cell interactions arise from the premise that *C*. *albicans* is a pathogen. In the vast majority of the human population however, *C*. *albicans* is simply a commensal. This leads to the fundamental question, what distinguishes *C*. *albicans* in the commensal state compared with the pathogenic state? One possibility is that *C*. *albicans* is inherently pathogenic. In this model, at low fungal cell density, manifestation of disease is sub-clinical and *C*. *albicans* is considered a benign agent; at high cell density disease, is readily apparent and *C*. *albicans* is considered a pathogen. However, when low concentrations of *C*. *albicans* are added to primary epithelial cells, *C*. *albicans* proliferate and form microcolonies on the surface of the epithelial cells without overt epithelial cell disruption [11], suggesting that ‘inherent pathogenicity’ is not sufficient to explain the observed difference between the commensal and pathogenic states. Another possibility is that the host itself, specifically epithelial cell responses contribute to the transition to the pathogenic state. For example, at low fungal cell density, epithelial cells may not respond to *C*. *albicans*. However, at high cell density, epithelial cells may respond in a way that elicits the expression of pathogenic traits in *C*. *albicans*. Indeed, a major area of current study is how the innate immune system distinguishes beneficial or commensal microbes from pathogens.

Here, we used the human FaDu epithelial cell line model to study epithelial cell responses to *C*. *albicans*. We found that some epithelial cell transcriptional responses are dependent on the dose of *C*. *albicans* used to initiate infection, whereas other responses are dose-independent. We found that members of the dual specificity phosphatase (DUSP) family are induced in response to *C*. *albicans* and that this induction is clearly linked to the activation of MAP kinases. These studies further define the epithelial cell response to *C*. *albicans* and to address the host response to a normal component of human flora.

## Materials and Methods

### Media and Growth Conditions

FaDu cells (ATCC, cat #30–2003) were routinely grown in MEM medium containing 10% FBS and 500 U/ml penicillin, 500 μg/ml streptomycin (Pen/Strep), and 1.25 μg/ml amphotericin B at 37°C in 5% CO_2_. For infection studies with fungi, amphotericin B was omitted from the tissue culture medium.

*C*. *albicans* strains (DAY185—wild-type, DAY25—*rim101Δ/Δ*, and DAY1175—*efg1Δ/Δ cph1Δ/Δ*) and *S*. *cerevisiae* (DAY414) were routinely grown in YPD (2% Bacto-peptone, 1% yeast extract, 2% dextrose) at 30°C [12–14]. For infections studies, *C*. *albicans* were grown in YPD overnight at 30°C. Cells were diluted 1:100 in DMEM, sonicated, and counted on a hemacytometer. *C*. *albicans* were diluted as appropriate in pre-warmed in DMEM containing 10% FBS and 200 units/ml of penicillin and streptomycin prior to adding to host cells.

### RNA Purification

Total RNA was isolated using TRIzol^R^ reagent (Invitrogen) and treated with DNAse I (Roche) for 30 minutes at 25°C. RNA was purified using an RNeasy mini column (Qiagen, Valencia CA) and eluted with nuclease free ddH_2_O. RNA concentration was determined using the NanoDrop ND-1000 Spectrophotometer (NanoDrop Technologies, Inc., Wilmington, DE).

### Microarrays

FaDu cell gene expression profiles were determined in triplicate with and without *C*. *albicans* using GeneChip^R^ HG-U133A 2.0 Microarrays (Affymetrix, Santa Clara CA). FaDu cells were split into 100 mm cell culture petri dishes and grown to ~95% confluency. Fresh DMEM containing 10%FBS and Pen/Strep with or without *C*. *albicans* was added and incubated as described in the text.

5–8 μg of purified RNA was processed using Affmetrix automated GeneChip^R^ standard protocols by the Microarray Facility at the Biomedical Genomics Center (U of MN). Gene expression values were determined using the Affymetrix Microarray Analysis Suite (MAS) 5.0 and chip-to-chip normalization with LOWESS done using Genedata Expressionist Pro 4.5. The Paired T-test was used to compare the control and test groups and genes differentially expressed ≥1.5 with a *P* value < 0.05 were examined.

### Real Time RT-PCR

FaDu cell gene expression changes by real time RT-PCR were determined in triplicate using the Taqman^R^ system (Applied Biosystems). FaDu cells were split into 100 mm cell culture petri dishes and grown to ~95% confluency. Fresh MEM containing 10%FBS and Pen/Strep with or without *C*. *albicans* or *S*. *cerevisiae* was added and incubated as described in the text. At each time point, RNA was purified, as described above, and 2.5 μg was used to generate cDNA using AMV Reverse Transcriptase and Recombinant RNasin®Plus Ribonuclease Inhibitor (Promega).

Real time RT-PCR was performed using 1/1944^th^ of the cDNA reaction mixture in 96 well format using a Bio-Rad IQ5 Real Time PCR Detection System (Bio-Rad) with TaqMan^R^ Gene Expression Assay primer and probe sets (Table 1). Each sample was run in triplicate for each gene of interest and the experiment was independently repeated two times.

**Table 1:** Primer sets used for RT-PCR.

Gene name	Assay ID
HPRT	Hs99999909_m1
DUSP 1	Hs00610256_g1
DUSP 6	Hs00169257_m1
Interleukin 1 alpha	Hs00174092_m1
Interleukin 6	Hs00174131_m1
Interleukin 8	Hs00174103_m1
Interleukin 24	Hs01114274_m1
SERPIN E1	Hs00167155_m1

### Protein Purification and Analysis

FaDu cells monolayers were placed on ice and washed 2x with ice cold PBS containing protease inhibitor cocktail (1ng/ml leupeptin, 2ng/ml pepstatin, 100ng/ml aprotinin, and 1mM PMSF). 0.7ml of lysis buffer (40 mM Tris pH 6.8, 1% SDS, 10% Glycerol, 2% β-mercaptoethanol, 0.01% Bromophenol Blue) was added and cells removed from the tissue culture plate using a cell scraper. Cells were transferred to a 2 ml screw capped tube, boil 5 minutes and stored at -80°C.

For Western blots, protein samples were thawed and boiled for 5 minutes and separated by 10% SDS-PAGE. Gels were transferred to nitrocellulose (Millipore Corp., Billerica, MA) and stained with 0.1% Ponceau S in 5% acetic acid to qualitatively assess loading and transfer. The blots were washed with ddH_2_O and blocked in TBS-T (50 mM Tris pH 7.6, 150 mM NaCl, 0.05% Tween-20) containing either 5% non-fat dairy milk or 5% BSA at 4°C. 1:1000–1:10,000 dilution of primary antibody, in blocking solution, was added according to manufacturer specifications and incubated overnight at 4°C or for 1 hour at 23°C. Primary antibodies used in this study are ⍺-Phospho-p42/44 MAPK (ERK1/2) and ⍺-phospho-JNK1/2 (Invitrogen) and ⍺-GAPDH (Cell Signaling Tech). The blots were washed 3 times with TBS-T at 23°C for 5 minutes and then 1:5000 HRP-donkey anti-rabbit IgG (Amersham Biosciences) was added for 1 hr at 23°C in 5% non-fat dairy milk in TBS-T. Blots were washed 3 times with TBS-T at 23°C for 5 minutes. Blots were treated with ECL Western Blotting Detection Reagents (Amersham Biosciences) and exposed to film. Films were analyzed using ImageJ version 1.38 (NIH) and bands normalized to GAPDH.`

## Results

### FaDu Cell Responses to *C*. *albicans*

*C*. *albicans* is part of the normal gastro-intestinal flora. To understand the host-pathogen interaction, it is essential to identify relevant host markers of pathogenesis. Thus, we used a human epithelial cell tissue culture model to identify transcriptional changes associated with *C*. *albicans* infection. Transcriptional profiling experiments were conducted using the FaDu cell line, an immortalized epithelial-like cell line derived from the oral-pharyngeal cavity of a male patient [15]. We chose this cell line as it is routinely used to study *C*. *albicans*-mediated host cell damage [4, 16]. Infection with high concentrations of *C*. *albicans* leads to rapid killing of epithelial cells [7, 17], yet we found that infection with low concentrations of *C*. *albicans* leads to proliferation on the surface of the epithelial cells without causing overt damage [11]. These results suggest that epithelial cell-*C*. *albicans* interactions vary depending on fungal concentration. We tested this idea by analyzing epithelial cell transcriptional responses following infection with a low or a high dose of *C*. *albicans*.

A 95% confluent monolayer of FaDu cells was infected for 6 hours with either 2.0 x 10^4^ cells/ml (low dose) or 2.5 x 10^6^ cells/ml (high dose) of wild-type *C*. *albicans* and epithelial cell transcriptional responses determined using Affymetrix gene chips. We chose the 6 hours time point to maximize the interaction time between host and microbe to allow host transcriptional responses to occur before *C*. *albicans*-mediated lethality is observed [17]. FaDu cells infected with a low dose of wild-type *C*. *albicans* differentially expressed 90 genes. Of these, 44 genes were down-regulated ≥ 1.5-fold and 46 genes were up-regulated ≥ 1.5-fold compared to uninfected controls (S1 Table). However, FaDu cells infected with a high dose of *C*. *albicans* differentially expressed 702 genes, of which, 309 were down-regulated ≥ 1.5-fold and 393 were up-regulated ≥ 1.5-fold compared to uninfected controls S2 Table.

**Fig 1:**
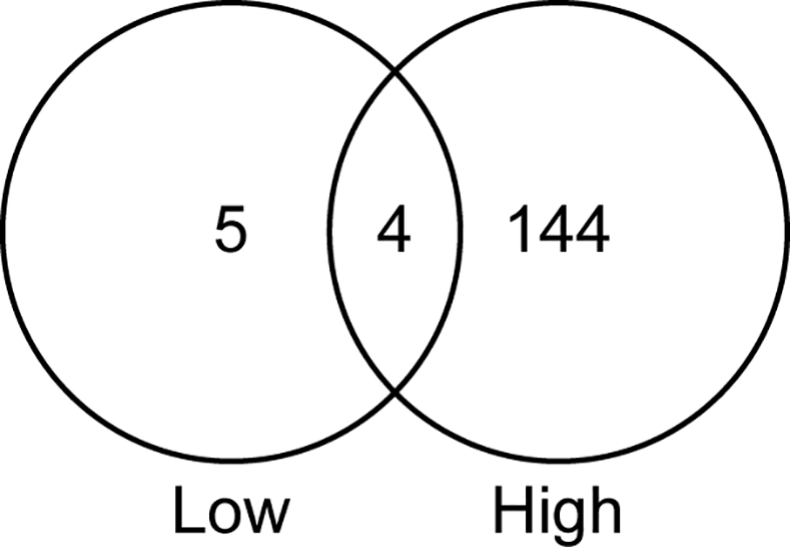
Differentially expressed host genes in response to differing doses of *C*. *albicans*. VENN diagram of genes differentially expressed in epithelial cells in response to a low and a high dose of *C*. *albicans*.

The high dose of *C*. *albicans* elicited a greater transcriptional response from the epithelial cells than the low dose of *C*. *albicans*. However, we were surprised to find that only ~25% (21/90) of the genes differentially expressed in response to the low dose of *C*. *albicans* were also differentially expressed in response to a high dose of *C*. *albicans* (Table 2). Of these 21 genes, 14 genes were induced > 2 fold and one gene, F5, was repressed > 2 fold in response to the high dose of *C*. *albicans* compared to a low dose of *C*. *albicans* (Table 2). One possibile explanation for these results is that they are due to weak induction under one condition that is not apparent in the other condition. However, three genes (SLC26A3, MFAP5, and TACR1) are induced > 5 fold in response to a low dose, but are not observed among the genes induced in response to a high dose of *C*. *albicans*. In fact using a > 3 fold change in gene expression revealed that of nine genes differentially expressed in epithelial cells in response to a low dose of *C*. *albicans*, only four genes were also differentially expressed in response to a high dose of *C*. *albicans* (Fig 1). These results demonstrate that while 21 host genes show potential dose-dependent expression, many of the observed transcriptional responses are not readily explained by a dose-dependent model.

**Table 2:** Common Epithelial Cell Responses to High and Low Dose *C*. *albicans*.

Expression Pattern	Affymetrix ID	Gene	Gene Product	Low dose	High dose	High/Low Ratio
HIGH ↑/LOW ↑	215459_at	CTNS	cystinosis, nephropathic	1.6	1.6	1.0
	201041_s_at	DUSP1	dual specificity phosphatase 1	3.0	52.1	17.4
	209189_at	FOS	v-fos fbj murine osteosarcoma viral oncogene homolog	6.2	113.9	18.3
	202768_at	FOSB	fbj murine osteosarcoma viral oncogene homolog b	4.5	35.1	7.7
	201631_s_at	IER3	immediate early response 3	1.6	18.6	12.0
	202859_x_at	IL8	interleukin 8	2.3	38.4	16.8
	201466_s_at	JUN	v-jun sarcoma virus 17 oncogene homolog	1.8	11.6	6.4
	213120_at	KIAA0701	kiaa0701 protein	2.0	3.1	1.6
	205269_at	LCP2	lymphocyte cytosolic protein 2	8.8	7.5	0.9
	202340_x_at	NR4A1	nuclear receptor subfamily 4, group a, member 1	1.7	3.6	2.1
	217996_at	PHLDA1	pleckstrin homology-like domain, family a, member 1	1.5	17.2	11.3
	204286_s_at	PMAIP1	phorbol-12-myristate-13-acetate-induced protein 1	1.9	5.3	2.7
	204748_at	PTGS2	prostaglandin-endoperoxide synthase 2	2.1	13.8	6.6
	212845_at	SAMD4A	sterile alpha motif domain containing 4a	3.3	12.2	3.7
	216236_s_at	SLC2A3	solute carrier family 2, member 3 or 14	1.6	6.3	4.0
	202241_at	TRIB1	tribbles homolog 1	1.5	8.1	5.3
	210513_s_at	VEGFA	vascular endothelial growth factor	1.5	3.7	2.5
HIGH ↓/LOW ↓	204714_s_at	F5	coagulation factor v (proaccelerin, labile factor)	-2.2	-4.5	2.1
	214836_x_at	IGKC—IGKV1-5	immunoglobulin kappa constant—immunoglobulin kappa variable 1–5	-2.1	-3.8	1.8
HIGH ↑/LOW ↓	204897_at	PTGER4	prostaglandin e receptor 4 (subtype ep4)	1.6	-2.7	NA
HIGH ↓/LOW ↑	204273_at	EDNRB	endothelin receptor type b	-4.4	1.7	NA

To gain insights into the likely host cell responses to *C*. *albicans*, we analyzed the transcriptional profiling data via gene ontogeny (GO) analysis [18, 19] (Tables 3 and 4). Epithelial cells exposed to either a low or a high dose of *C*. *albicans* induced genes associated with apoptosis, the inflammatory response, and the response to stress (*P* value < 0.01). However, epithelial cells exposed to a high dose of *C*. *albicans* also induced genes associated with chemotaxis and host-pathogen linked responses (*P* value < 0.001) (Table 3). Additionally, epithelial cells exposed to a high dose of *C*. *albicans*, repressed genes associated with the cell cycle and macromolecular synthesis (Table 4). No GO categories were repressed with statistical significance in response to the low dose of *C*. *albicans*.

**Table 3:** Relevant GO categories of Induced Genes.

		Low Dose 1.5 Up		High Dose 1.5 Up	
GO ID	Term	#	*P*	#	*P*
GO:0001525	Angiogenesis	0	NA	21	5.6E-11
GO:0006915	Apoptosis	7	6.8E-03	55(1*)	9.4E-14
GO:0007049	Cell cycle	4	p > 0.05	42(4)	8.7E-06
GO:0008283	Cell proliferation	4	p > 0.05	60(4)	1.5E-16
GO:0007267	Cell-cell signaling	0	NA	32	7.7E-05
GO:0006955	Immune response	0	NA	37	1.0E-03
GO:0006954	Inflammatory Response	5	4.2E-03	18(3)	4.3E-04
GO:0006935	Chemotaxis	0	NA	15	6.1E-06
GO:0006950	Response to stress	8	8.4E-03	55(5)	1.6E-08
GO:0009611	Response to wounding	5	1.5E-02	33(3)	2.5E-09
GO:0007165	Signal transduction	15	2.3E-02	128(7)	9.2E-08

* #s in parentheses equal the number genes in common with the low dose.

NA not applicable

**Table 4:** Relevant GO categories of Repressed Genes.

		Low Dose 1.5 Down		High Dose 1.5 Down	
GO ID	Term	#	*P*	#	*P*
GO:0022403	Cell Cycle Phase	0	NA	26	1.1E-10
GO:0043283	Biopolymer Metabolic Process	13	p > 0.05	125(1*)	7.4E-10
GO:0006139	Nucleobase, Nucleoside, Nucleotide and Nucleic Acid Metabolic Process	10	p > 0.05	99(1)	2.6E-08
GO:0043170	Macromolecule Metabolic Process	17	p > 0.05	142(1)	2.8E-06

* #s in parentheses equal the number genes in common with the low dose.

NA not applicable

Innate inflammatory responses are essential in controlling *C*. *albicans* infections, thus we more closely analyzed the genes in the ‘inflammatory response’ GO category (Table 5). The low dose of *C*. *albicans* caused the specific induction of SERPIN A3, and TACR1; both low and high doses induced FOS, IL-8, and PTGS2 (COX-2). FOS, a component of the AP-1 transcription factor, regulates IL-8 and PTGS2 expression [20, 21]. The high dose of *C*. *albicans* induced fifteen additional genes, including the chemokines CCL20, CXCL1, CXCL2, CXCL3, and CXCL12 as well as IL-1⍺, IL-1β, and IL-6. IL-8, CCL20, CXCL1, and CXCL3 function in neutrophil recruitment and it is widely recognized that neutrophils are potent anti-Candida leukocytes. CXCR4, the CXCL12 receptor, is also induced suggesting the possibility an autocrine feedback pathway. Several of these host cell responses to *C*. *albicans*, such as IL-8 induction, have been reported previously, providing independent validation for the new responses identified here.

**Table 5:** Inflammatory Response Genes.

			Low Dose		High Dose	
Affymetrix ID	Gene	Gene product	*Average*	*P*	Average	*P*
208048_at	TACR1	tachykinin receptor 1	5.4	0.041	ND	NA
202376_at	SERPINA3	serpin peptidase inhibitor, clade a, member 3	1.9	0.014	ND	NA
209189_at	FOS	v-fos fbj murine osteosarcoma viral oncogene homolog	6.3	0.01	113.9	0.016
202859_x_at	IL8	interleukin 8	2.3	0.009	38.4	0.003
204748_at	PTGS2	prostaglandin-endoperoxide synthase 2	2.1	0.04	13.8	0.003
205476_at	CCL20	chemokine (c-c motif) ligand 20	ND	NA	33.8	0.004
211919_s_at	CXCR4	chemokine (c-x-c motif) receptor 4	ND	NA	17.2	0.048
204470_at	CXCL1	chemokine (c-x-c motif) ligand 1	ND	NA	9.7	0.017
209774_x_at	CXCL2	chemokine (c-x-c motif) ligand 2	ND	NA	7.1	0.011
201925_s_at	CD55	cd55 antigen, decay accelerating factor for complement	ND	NA	6.3	0.001
207850_at	CXCL3	chemokine (c-x-c motif) ligand 3	ND	NA	5.3	0.033
205207_at	IL6	interleukin 6 (interferon, beta 2)	ND	NA	5.1	0.002
210118_s_at	IL1⍺	interleukin 1, alpha	ND	NA	4.8	0.006
205289_at	BMP2	bone morphogenetic protein 2	ND	NA	3.2	0.002
201531_at	TTP, Zfp-36	zinc finger protein 36, c3h type, homolog (mouse)	ND	NA	3.2	0.002
203923_s_at	CYBB	cytochrome b-245, beta polypeptide	ND	NA	3.0	0.016
205067_at	IL1β	interleukin 1, beta	ND	NA	2.6	0.011
209687_at	CXCL12	chemokine (c-x-c motif) ligand 12	ND	NA	1.9	0.008
207655_s_at	BLNK	b-cell linker	ND	NA	1.8	<0.001
217930_s_at	TOLLIP	toll interacting protein	ND	NA	1.6	0.025

ND—not detected

NA—not applicable

We noted that 6 members of the DUSP gene family were induced in response to *C*. *albicans* infection. DUSP1 was induced by both the low and high doses of *C*. *albicans*; DUSP4, 5, 6, 7, and 10 were induced in response to a high dose of *C*. *albicans*. The DUSP gene family encodes protein phosphatases that inactivate MAP kinases. DUSP proteins regulate inflammatory responses and mice lacking DUSP1 have increased cytokine expression and increased sepsis in response to endotoxin [22, 23].

### Host Responses Are Dependent on the Ability of *C*. *albicans* to Cause Damage

Our transcriptional profiling studies suggest that the transcriptional responses of FaDu cells to *C*. *albicans* are not all dose-dependent (S1 and S2 Tables and Tables 3 and 4). Thus, we posited a different model where a subset of host responses is dependent on the ability of *C*. *albicans* to induce damage. We predicted that when *C*. *albicans* is not promoting damage, the host response resembles that observed in our low dose studies. To address this idea, we analyzed host responses after exposure to a high dose of mutant *C*. *albicans* cells that have reduced abilities to cause host cell damage. Specifically, we used the *rim101Δ/Δ* mutant, which elicits ~5% killing, and the *efg1Δ/Δ cph1Δ/Δ* mutant, which does not promote epithelial cell lysis [4, 7]. We also analyzed *S*. *cerevisiae* as a control, which does not adhere to nor promote epithelial cell damage [24].

**Fig 2:**
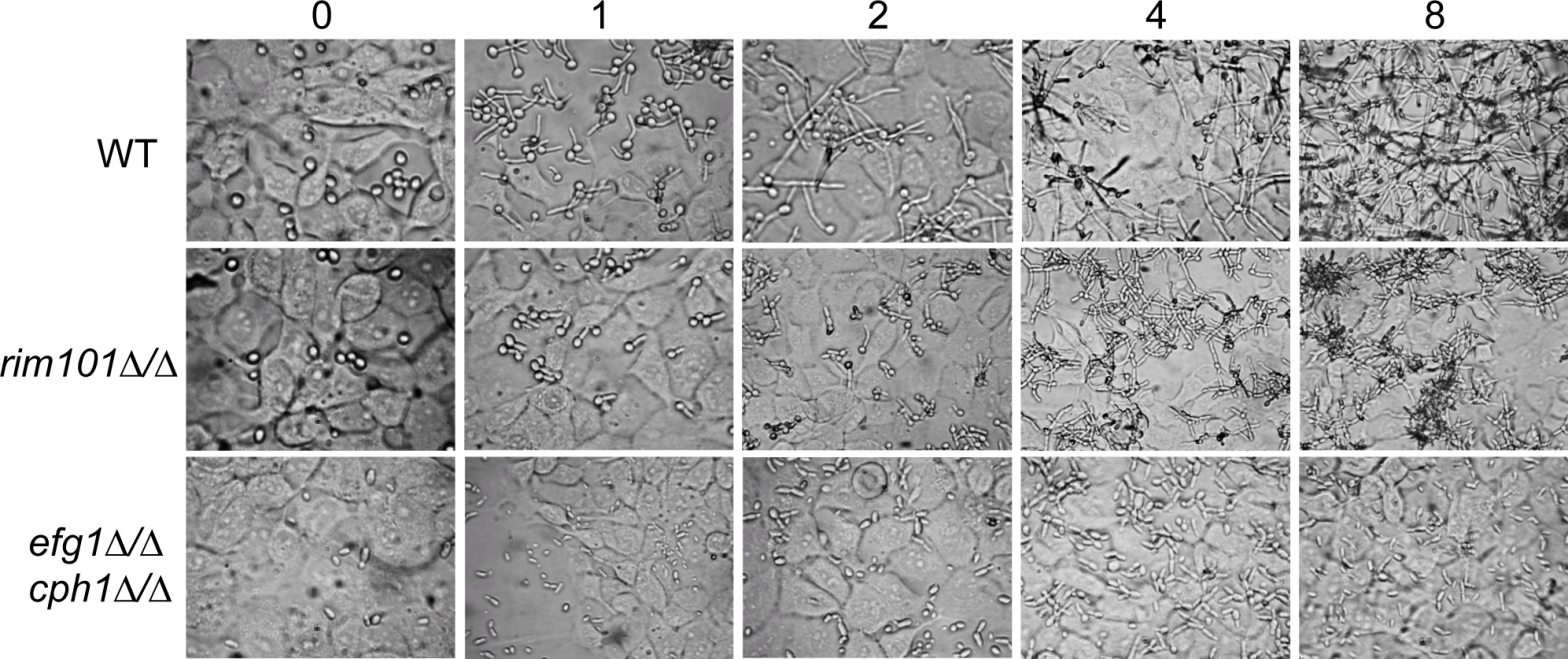
Growth of *C*. *albicans* on epithelial cells. Wild-type, *rim101Δ/Δ*, and *efg1 cph1Δ/Δ* strains were co-cultured with FaDu epithelial cell monolayers at 37°C in a 5% CO_2_ incubator. At the indicated times post-infection cells were photographed.

We considered that differences in epithelial transcriptional responses to the *C*. *albicans* mutants could be due to kinetic differences associated with the interaction between the mutants and epithelial cells and not a fundamental difference in the interaction itself. To control for this, we used real time RT-PCR to compare gene expression changes over 32 hours (compared with the 6 hour snap shot used in the original microarrays). We were also concerned that the differences in the yeast-hyphal morphological transition, which is critical for pathogenesis, would promote distinct host cell responses [25]. We found that wild-type yeast cells germinated hyphae within 1 hour post-infection (Fig 2). The hyphae continued to grow over the epithelial cells and formed a hyphal mat within 8 hours. The *rim101Δ/Δ* mutant also germinated to form hyphae within 1 hour post-infection. However, unlike wild-type *C*. *albicans*, the *rim101Δ/Δ* mutant hyphae grew more slowly and only began branching by 4 hours post-infection. Eight hours post-infection, the *rim101Δ/Δ* mutant had not formed a complete mat, likely due to the shorter branching hyphae. The *efg1Δ/Δ cph1Δ/Δ* mutant grew in the yeast form throughout the 32 hour incubation and unlike the wild-type and *rim101Δ/Δ* cells, the *efg1Δ/Δ cph1Δ/Δ* mutant was non-adherent and could be dislodged from the epithelial cells with gentle agitation. Thus, if a host response is due to *C*. *albicans* morphology, the *rim101Δ/Δ* mutant should show a kinetic defect due to the less robust hyphal formation and the same effect should be absent in the *efg1Δ/Δ cph1Δ/Δ* mutant and *S*. *cerevisiae*, which do not form hyphae.

**Fig 3:**
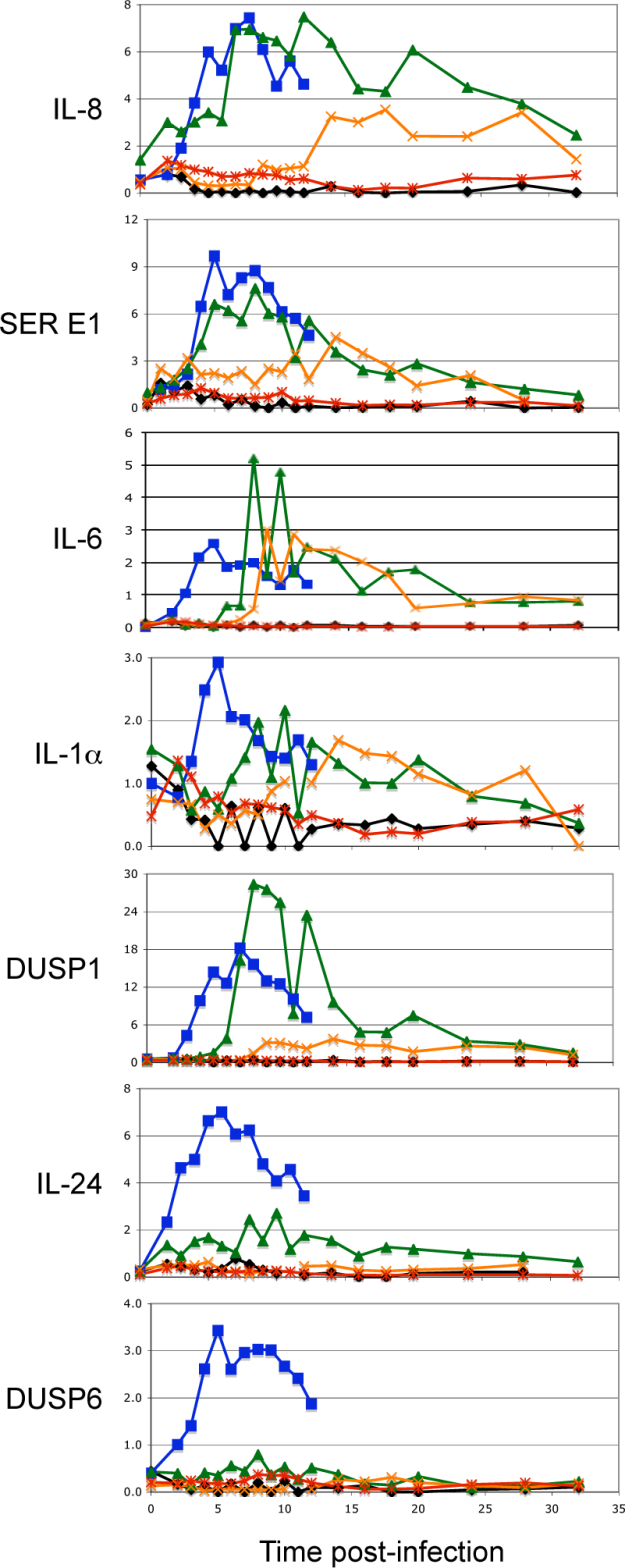
Effect of *C*. *albicans* mutations on host transcriptional responses. RT-PCR for IL-8, SERPIN E1 (SER E1), IL-6, IL1⍺, DUSP1, IL-24, and DUSP6 transcription, expressed in relative ng (y-axis). cDNA derived from mRNA purified from FaDu epithelial cell monolayers infected with wild-type *C*. *albicans* (■), *rim101Δ/Δ* (▲), mutant cells, *efg1 cph1Δ/Δ* (×) mutant cells, *S*. *cerevisiae* (*), or non-infected (◆) at defined time points (x-axis). A single time course experiment is shown, but analogous results were obtained in two replicate experiments.

IL-8 and SERPIN E1 were induced 3 hours post-infection with wild-type *C*. *albicans* and reached a maximal level of induction 8 hours post-infection (Fig 3). Similar results were observed when epithelial cells were infected with the *rim101Δ/Δ* mutant. However, when epithelial cells were exposed to a high dose of the *efg1Δ/Δ cph1Δ/Δ* mutant, IL-8 and SERPIN E1 expression were induced ~14 hours post-infection and only reached ~50% of the level observed with exposure to either the wild-type or *rim101Δ/Δ* strains. *S*. *cerevisiae* did not induce IL-8 or SERPIN E1 expression, even after 32 hours of co-culture.

IL-6 and IL-1⍺ were induced 5 hours post-infection with wild-type *C*. *albicans* cells. However, when epithelial cells were infected with a high dose of the *rim101Δ/Δ* mutant, IL-6 and IL-1⍺ were expressed ~8–9 hours post-infection. Infection of the epithelial cells with the *efg1Δ/Δ cph1Δ/Δ* mutant induced IL-6 and IL-1⍺ ~ 9 and 14 hours post-infection respectively. *S*. *cerevisiae* did not induce IL-6 and IL-1⍺ expression during the 32 hour incubation.

DUSP1, IL-24, and DUSP6 were induced 2–3 hours post-infection with the high dose of wild-type *C*. *albicans* and reached a maximal level of induction ~6 hours post-infection. DUSP1 was induced in epithelial cells ~6 hours post-infection and reached a maximal level after 8 hours following infection with the *rim101Δ/Δ* mutant. However, the *rim101Δ/Δ* mutant did not induce IL-24 or DUSP6 expression. Neither the *efg1Δ/Δcph1Δ/Δ* mutant nor *S*. *cerevisiae* induced DUSP1, IL-24, or DUSP6 expression over the 32 hours of co-culture.

In total, wild-type *C*. *albicans* cells rapidly induced host cell expression of all tested genes and reached a maximum within 5 hours post-infection. The *rim101Δ/Δ* mutant showed similar induction (IL-8), delayed induction (DUSP1), or no induction (DUSP6) of host responses; the *efg1Δ/Δcph1Δ/Δ* mutant showed delayed (IL-8) or no induction (DUSP1) of host responses. These results collectively support the idea that a subset of the induced host cell responses are not fungal dose-dependent, but may instead be dependent on host cell damage.

### Epithelial Cells Activate MAP Kinases upon Exposure to *C*. *albicans*

In response to pathogenic microbes, epithelial cells stimulate mitogen activated protein (MAP) kinases, which promote transcription factor activity. Our transcriptional profiling experiments suggest that MAP kinases are activated via phosphorylation in response to *C*. *albicans*. To directly test this possibility, we analyzed MAP kinase phosphorylation following exposure to wild-type and mutant *C*. *albicans* and *S*. *cerevisiae*.

**Fig 4:**
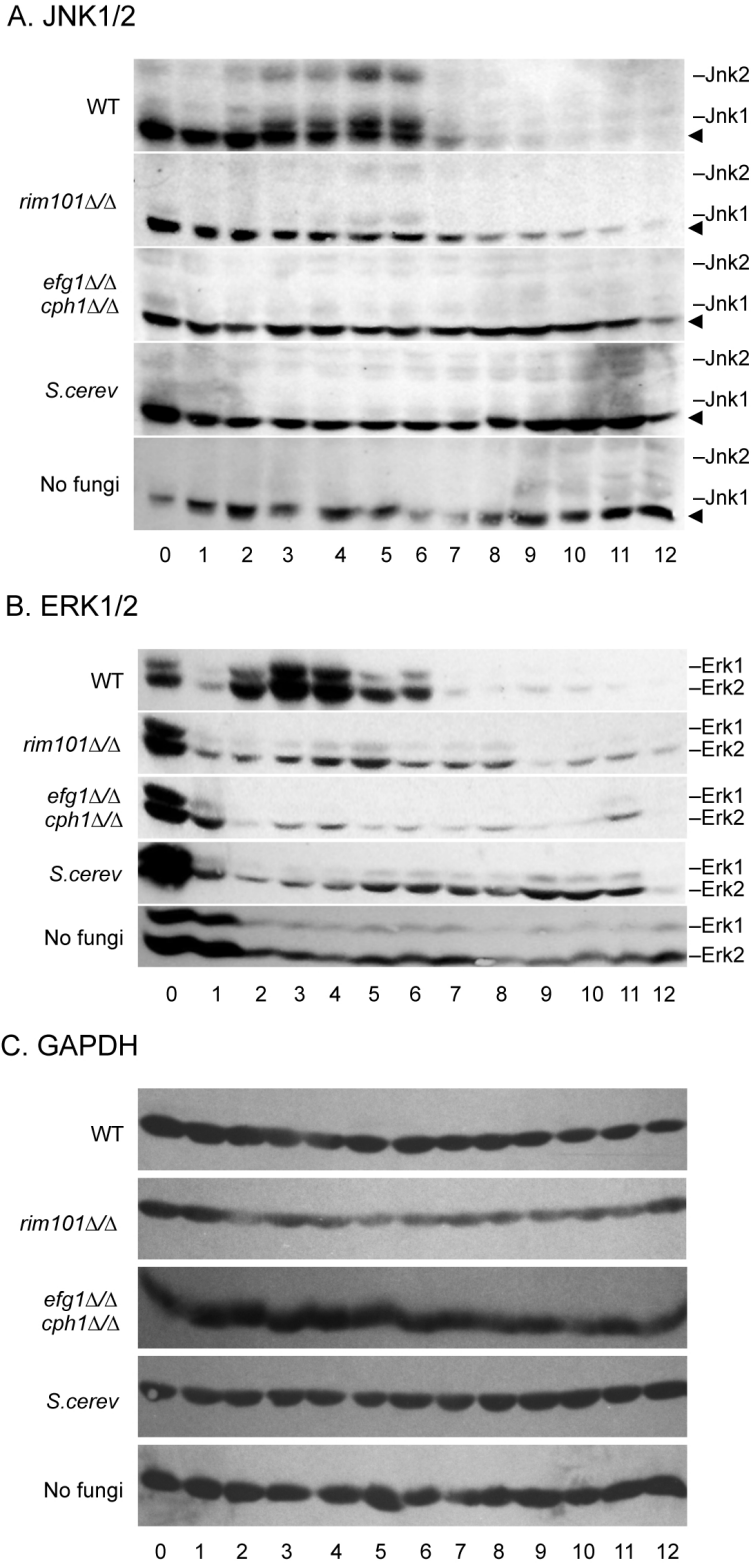
MAP kinase activation stimulated by *C*. *albicans* mutants. Total protein was purified from infected or non-infected FaDu epithelial cell monolayers at defined time points. Proteins were separated by SDS-PAGE and western blots probed with anti-phopho-JNK1/2 (A) or anti-phospho-ERK1/2 (B). A cross-reactive background band (arrowhead) was often observed with the anti-phospho-JNK1/2 antibody. Protein loading was normalized by detection of GAPDH (C). Results shown are representative of three independent experiments.

The JNK1/2 MAP kinases were phosphorylated 3 hours post-infection with wild-type *C*. *albicans* (Fig 4A). JNK1/2 phosphorylation was maintained until 7 hours post-infection when phosphorylation was reduced to background levels. JNK1/2 phosphorylation was delayed and only observed 4–6 hours following infection with the *rim101Δ/Δ* mutant. JNK1/2 phosphorylation was also markedly reduced during infection with the *rim101Δ/Δ* mutant when compared to infection by wild-type *C*. *albicans*. No JNK1/2 phosphorylation was observed when epithelial cells were infected with the *efg1Δ/Δ cph1Δ/Δ* mutant nor with *S*. *cerevisiae*. These results demonstrate that robust JNK1/2 phosphorylation occurs in response to wild-type *C*. *albicans* and is reduced or absent in response to less pathogenic strains.

The ERK1/2 MAP kinases were phosphorylated 2 hours post-infection with wild-type *C*. *albicans* and the phosphorylated state was maintained until 7 hours post-infection (Fig 4B). Little ERK1/2 phosphorylation was observed following infection with the *rim101Δ/Δ* mutant and no ERK1/2 phosphorylation was observed following infection with the *efg1Δ/Δ cph1Δ/Δ* mutant nor with *S*. *cerevisiae*. Similar results were observed for p38 phosphorylation (data not shown). These results confirm that wild-type *C*. *albicans* promote robust MAP kinase activation.

## Discussion

The ability of the immune system to distinguish between commensal and pathogenic microbes is critical to host survival. As the primary cell type at the interface between the external environment and internal host tissues, epithelial cells are key players in this role. Inappropriate responses to commensal organisms at the mucosa by epithelial cells can lead to chronic inflammation with clinical manifestations [26, 27]. Conversely, if commensals are completely ignored, then those with disease causing potential, such as *C*. *albicans*, can overgrow and cause disease. The current paradigm is that the host is aware of, but not responsive to, commensal microbes.

Here, we found that the FaDu epithelial-like cell line does respond to a dose of *C*. *albicans* that causes little if any damage (Tables 2–5). When compared to a dose of *C*. *albicans* that causes extensive damage, these low-dose responses were both quantitatively diminished and qualitatively distinct. While some of these responses did appear to be dose-dependent, others were not and appeared associated with the yeast-hyphal transition and/or ability of *C*. *albicans* to cause damage. Induction of epithelial responses was correlated with phosphorylation of epithelial MAP kinases and involved induction of the DUSP protein family.

The low or ‘commensal’ dose of *C*. *albicans* used in these studies led to the induction of a small number of genes involved in stress and inflammatory responses, such as IL-8. However, most genes involved in inflammatory responses were not induced, suggesting that the epithelial cells have recognized the *C*. *albicans* cells and entered into a sentinel mode: awareness without overt changes in homeostatic processes. The high or ‘pathogenic’ dose of *C*. *albicans* led to the induction of many genes involved in stress and inflammatory responses, as well as genes involved in other immune responses and chemotaxis (Table 3). These results indicate that the epithelial cells have entered into a defensive mode: potentiating immunological responses, recruiting effector cells, and halting other activities including growth. Where host responses to a low or high dose of *C*. *albicans* overlap, such as with FOS, JUN, and IL-8 expression, the high dose of *C*. *albicans* generally elicited a greater effect on gene expression than the low dose. This idea of epithelial cells having sentinel and defensive roles has been suggested previously [26, 27], including for *C*. *albicans* [28].

One explanation for the change from a sentinel state to a more active defensive state is the number of *C*. *albicans* cells interacting with the epithelial surface. Indeed, of the host cell genes differentially expressed in response to *C*. *albicans*, ~90% (including those only induced in response to the high dose of *C*. *albicans*) could be explained using a dose-dependent model. The proximate causes underlying dose-dependent differences in host-*C*. *albicans* interactions are currently unknown and could be mediated by either the host or the pathogen. While most host responses are explained using a dose-dependent model, we have identified numerous host responses that are not. Furthermore, our analyses using a high dose of wild-type and mutant *C*. *albicans* suggest that many dose-dependent changes must be affected by more than just the absolute numbers of *C*. *albicans* cells. Two hours post-infection cell numbers among the wild-type, *rim101Δ/Δ*, and *efg1Δ/Δ cph1Δ/Δ* strains are similar (Fig 2), yet epithelial cells induced DUSP6 only in response to wild-type *C*. *albicans* (Fig 3). Neither mutant significantly induced DUSP6 over the 32 hour time course despite the fact that both mutants grew and reached concentrations much greater than that observed when DUSP6 was induced by wild-type *C*. *albicans*. Furthermore, in some cases the *rim101Δ/Δ* mutant elicited host responses that matched wild-type *C*. *albicans*, such as SERPIN E1, and in other cases the *rim101Δ/Δ* mutant elicited host responses that matched the *efg1Δ/Δ cph1Δ/Δ* mutant, such as IL-6. Thus, differences in *C*. *albicans* dose are not sufficient to explain many observed host responses.

Because *C*. *albicans* morphology can influence host responses [29], we also considered whether the morphogenetic differences observed in the various *C*. *albicans* mutants provide insight into host cell responses. However, morphogenetic differences did not appear sufficient to predict host cell responses. For example, if this model were correct, epithelial transcriptional responses to the high and low dose of wild-type *C*. *albicans* should be similar as *C*. *albicans* grow in the hyphal form on the surface of epithelial cells regardless of dose. Furthermore, as noted above the *rim101Δ/Δ* mutant formed hyphae with delayed kinetics compared to wild-type cells yet frequently did not show an associated host response, such as DUSP6 induction.

One *C*. *albicans* property that is associated with both dose and morphogenetic potential and predicts host cell responses is the ability to promote epithelial cell damage. In previous studies with the FaDu epithelial cell line, wild-type *C*. *albicans* elicited ~25% host cell killing, while the *rim101Δ/Δ* mutant elicited ~5% killing, and *efg1Δ/Δ cph1Δ/Δ* cells did not promote epithelial cell damage [4, 7]. While *S*. *cerevisiae* has not been studied in this specific model, it does not adhere to host cells, nor does it promote damage in other models [24]. Thus, with respect to the ability to kill epithelial cells in culture: wild-type > *rim101Δ/Δ* > *efg1Δ/Δ cph1Δ/Δ* = *S*. *cerevisiae*. This same relationship between fungal strain and epithelial damage in host cell responses was also observed for JNK1/2 phosphorylation: wild-type *C*. *albicans* promote more JNK1/2 phosphorylation than the *rim101Δ/Δ* mutant, which promotes more JNK1/2 phosphorylation than the *efg1Δ/Δ cph1Δ/Δ* mutant or *S*. *cerevisiae*. Since JNK1/2 phosphorylation occurs well before host cell death mediated by wild-type *C*. *albicans*, JNK1/2 phosphorylation predicts the gene expression changes observed in the FaDu epithelial cells in response to a high and low dose of *C*. *albicans* as well as to the high dose of *C*. *albicans* mutant strains. Based on these transcriptional studies, we propose a differential-interaction model where some interactions between epithelial cells and a high dose of *C*. *albicans* are qualitatively distinct from the interaction between epithelial cells and a low dose of *C*. *albicans*. Host cell killing is a qualitative distinction that distinguishes the high and low doses of *C*. *albicans* as well as the separate mutant strains studied here.

### Are These Studies Applicable to Human Invasive Infections?

One potential limitation of the transcriptional studies is that a single epithelial cell line was used raising the question of general applicability. However, the overlap between our analyses and other microbial-epithelial cell interaction studies suggests that our results are providing relevant data. First, our transcriptional results overlap well with those obtained by Barker et al. who identified endothelial cell responses to *C*. *albicans* and Liu et al. who used RNA-seq to identify epithelial and endothelial cell responses in *C*. *albicans* [30, 31]. Indeed, we observed NEDD9 up-regulation in response to a high dose of *C*. albicans, but not a low dose of *C*. *albicans* S2 Table, which Liu et al found to be important for host cell endocytosis of *C*. *albicans* hyphae [31]. We also find much overlap between our results and those of Howie et al. studying T84 human colonic epidermoid cell responses to *Neisseria gonorrhoeae*, including the induction of DUSP1, DUSP2, and DUSP5 [32]. Second, many of the transcriptional responses we observed are predicted if the MAP kinase cascades are activated, which we found to be the case. While we are confident that the FaDu cell line revealed host responses relevant to the *in vivo* scenario, the cell line does have limitations. For example, only restricted aspects of pathogenesis can be analyzed using this, or any other, cell line, including pathogen adherence and host cell damage. Furthermore, we have found that FaDu epithelial cells lack certain properties found in “normal” epithelial cells. When exposed to FaDu epithelial cells, *rim101Δ/Δ* mutants can germinate and grow as hyphae (Fig 2). However, *rim101Δ/Δ* mutants grow only in the yeast form in animal models [33]. Since the mammalian model is analogous to the human, as compared to an immortalized and transformed cell line, we infer that the inhibition of *rim101Δ/Δ* hyphal growth in the animal model is an attribute of epithelial cells that has been lost in the FaDu cell line.

## Supporting Information

S1 Table Genes differentially expressed ≥1.5 fold in epithelial cells in response to a low dose of *C*. *albicans*. (XLSX)

S2 Table Genes differentially expressed ≥1.5 fold in epithelial cells in response to a high dose of *C*. *albicans*. (XLSX)
